# Measures of Quality of Care for People with HIV: A Scoping Review of Performance Indicators for Primary Care

**DOI:** 10.1371/journal.pone.0136757

**Published:** 2015-09-28

**Authors:** Sharon Johnston, Claire Kendall, Matthew Hogel, Meaghan McLaren, Clare Liddy

**Affiliations:** 1 Department of Family Medicine, University of Ottawa, Ottawa, Canada; 2 C.T. Lamont Primary Health Care Research Centre, Bruyère Research Institute, Ottawa, Canada; British Columbia Centre for Excellence in HIV/AIDS, CANADA

## Abstract

The healthcare of people with HIV is transitioning from specialty care to the primary healthcare (PHC) system. However, many of the performance indicators used to measure the quality of HIV care pre-date this transition. The goal of this work was to examine how existing HIV care performance indicators measure the comprehensive and longitudinal care offered in a PHC setting. A scoping review consisting of peer-reviewed and grey literature searches was performed. Two reviewers evaluated study eligibility and indicators in documents meeting inclusion criteria were extracted into a database. Indicators were matched to a PHC performance measurement framework to determine their applicability for evaluating quality of care in the PHC setting. The literature search identified 221 publications, of which 47 met inclusion criteria. 1184 indicators were extracted and removal of duplicates left 558 unique indicators. A majority of the 558 indicators fell under the ‘secondary prevention’ (12%) and ‘care of chronic conditions’ (33%) domains when indicators were matched to the PHC performance framework. Despite the imbalance, nearly all performance domains in the PHC framework were populated by at least one indicator with significant concentrations in domains such as patient-provider relationship, patient satisfaction, population and community characteristics, and access to care. Existing performance frameworks for the care of people with HIV provide a comprehensive set of indicators that align well with a PHC performance framework. Nonetheless, some important elements of care, such as patient-reported outcomes, are poorly covered by existing indicators. Advancing our understanding of how the experience of care for people with HIV is impacted by changes in health services delivery, specifically more care within the PHC system, will require performance indicators to capture this aspect of HIV care.

## Introduction

The health care needs of people living with HIV are evolving as HIV is managed as a chronic condition over increasingly longer lifespans [1]. Complex chronic conditions like HIV are challenging traditional health system structures [2] as they transition from solely within specialist care to increasingly managed within the primary healthcare (PHC) system [3,4]. Adding a primary care physician as part of the healthcare team for a person with HIV has been shown to improve both primary care and HIV-related outcomes [5].

Continuous measurement of transformative processes and outcomes, as well as relevant feedback to the different stakeholders contributing to change is an essential part of supporting system change [6]. Effective use of feedback to support health system change relies on valid, accepted, and feasible indicators [7]. In a complex adaptive system like our evolving health system, different stakeholders contributing to the transformation may need, or value, different information depending on the elements of performance they may influence [8,9].

The purpose of this study was to identify existing HIV performance indicators which would encompass the disease-specific metrics as well the comprehensive care measures of people with HIV receiving care in a PHC setting. We further sought to ascertain whether existing HIV- focused indicators reflected the range of goals, and resources available, for high quality care delivered within the PHC system. We conducted a scoping review of the literature to identify performance indicators that have been proposed or are in use within Canada and the United States since 2000 to measure the quality of HIV care. We then explored how well these performance indicators match to a primary healthcare performance framework. The ultimate goal was to identify existing performance indicators to populate a performance measurement framework to guide care improvements for people with HIV across the specialty and primary healthcare sectors in which they receive care.

## Methods

The scoping review was part of a research program to advance the care of people living with HIV [10], and was approved by the Ottawa Health Science Network Research Ethics Board. Our review was guided by the methodology developed by Arksey and O’Malley [11] to address the research question ‘Do existing performance indicators intended to evaluate the quality of HIV care measure the comprehensive and longitudinal care offered in a PHC setting to people living with HIV?’ We adapted the PRISMA methodology (see S1 File for the PRISMA checklist) to guide our review. No protocol for this study was published prior to undertaking this work.

### Scoping Literature Review

#### Identifying relevant studies

Medline and EMBASE databases were searched on July 15, 2013. A grey literature search was performed on July 16, 2013 using the Google and Google Scholar search engines. The full search strategy is described in S2 File. Results returned by the search engines were screened until the links no longer had relevance to HIV and/or performance measurement. This occurred on page 11 of the Google search and page 12 of the Google Scholar search. A snowball search strategy was also employed during the grey literature search, where URL links were followed to identify additional relevant results.

#### Selecting studies

Eligibility criteria required publications to a) contain performance measures and information on the development or source of those measures, b) be focused on the quality of healthcare for people with HIV, c) be published in or after the year 2000, d) reflect the US and/or Canadian healthcare systems, and e) be published in English. We did not include documents with a primary goal of reporting HIV related statistics, if there was not also a focus on the development or rational for the indicators, thus excluding such sources as population health reports.

Titles and abstracts of the publications were initially screened by a single reviewer (MH) to determine whether they met eligibility requirements. A second reviewer (SJ) independently reviewed a random selection of 10% of the results to ensure agreement in applying the inclusion and exclusion criterion.

#### Charting the data

Performance indicators from the publications meeting eligibility requirements were extracted into a database. The extracted information included the indicator, numerator and denominator information for each indicator, the publication in which it was extracted from, and the publication where the indicator originated. For publications that produced new indicators, the methodology used in the development of those indicators was also documented. This included whether guidelines were referenced (and which ones), whether a panel, committee, or workgroup was assembled to provide guidance (and the types of expertise contained on those panels), whether any validation of the indicators was documented, and other methodological information deemed relevant.

We grouped indicators covering the same process or outcome as similar as our interest was in the degree of comprehensiveness and match to different PHC performance domains. Accordingly, indicators were considered similar or covering the same process or outcome of care despite differences in qualifiers such as age of application or time frame within which a process should occur. For example, an indicator requiring mammograms every 2 years for women over 50 years of age was classified as similar to an indicator requiring mammograms every year for women over 40 years of age.

Collating, summarizing, and reporting the results. A PHC-specific performance framework was selected to apply a PHC lens and structure to organising the HIV performance indicators [12]. The PHC framework is broken into two overarching domains of care, the structural and the performance elements. The structural domain “includes the organizational and environmental features likely to influence primary care service delivery” [12]. The performance domain is comprised of aspects of health care service delivery, and technical quality of care which is defined as “the degree to which clinical procedures reflect current research evidence and/or meet commonly accepted standards for technical content or skill” [12]. This PHC framework was adapted to include a third segment under the ‘performance’ domain titled ‘patient reported outcomes’, which includes patient satisfaction, patient activation and empowerment, and patient engagement. Each indicator extracted was assigned to the most applicable domain within the adapted PHC-specific performance framework. Both MH and SJ reviewed and agreed on the matching of each indicator to the PHC performance domain.

## Results

### Sources of HIV Performance Measures

**Fig 1:**
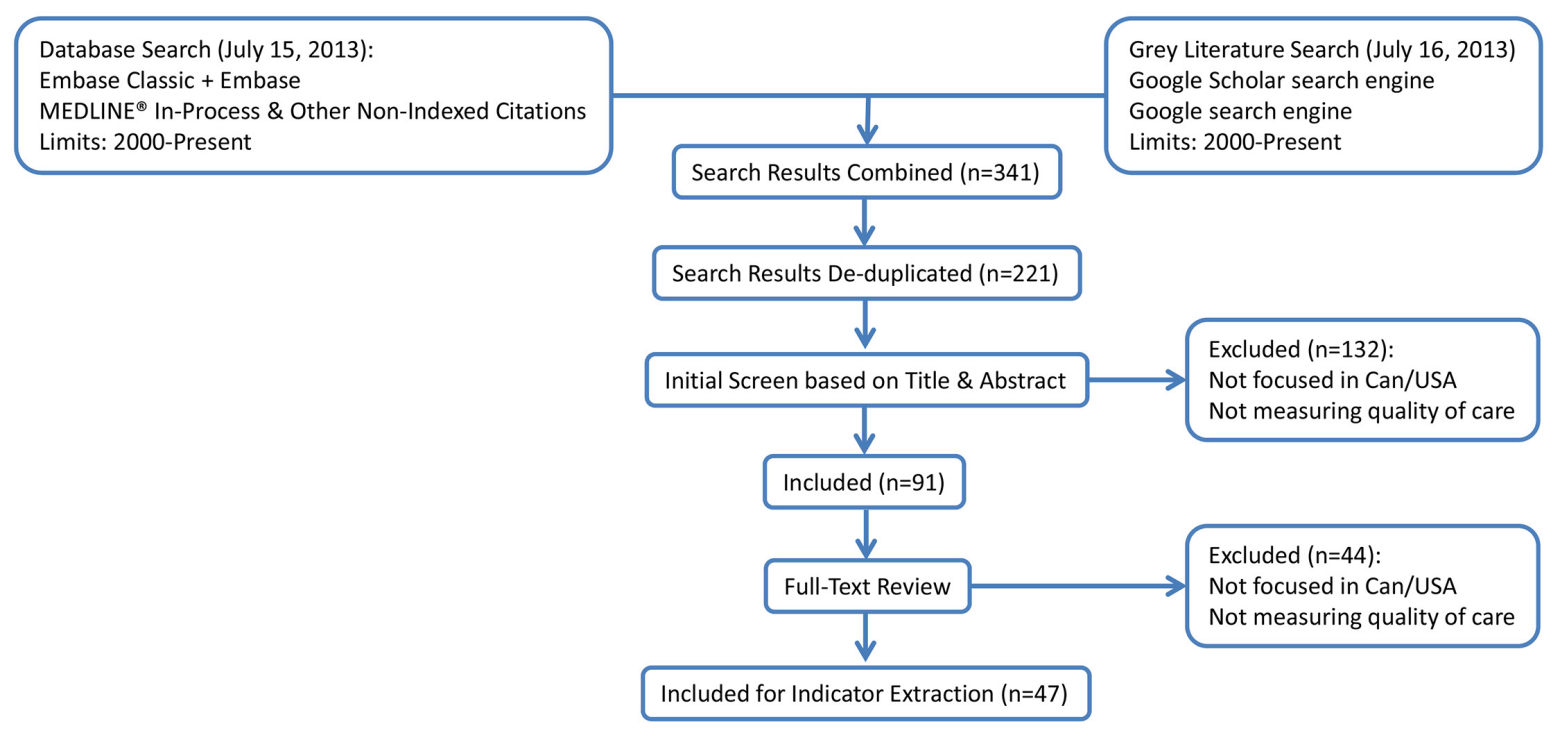
Flowchart describing the filtering of publications through the scoping review process.

The systematic review search strategy retrieved 196 unique items from the MEDLINE and EMBASE databases as well as 5 and 20 additional items using the Google Scholar and Google search engines, respectively. In total, the search generated 221 unique items from the databases and the grey literature. Iterative screening (Fig 1) resulted in the inclusion of 47 documents. The full list of included documents can be found in S3 File.

Thirty-one of the 47 included documents were obtained from the peer-reviewed literature while 16 were found in the grey literature. Eight of those 16 were reports, 6 were website repositories, and 2 were presentations published online. The documents obtained through database searches were produced predominantly from individuals affiliated with academic and/or research institutions, while those obtained from the grey literature were produced by government agencies and by HIV-specific organizations. There was a significant difference between the quantity and focus of indicators contained in documents retrieved from databases compared to those obtained through the grey literature. Documents from the grey literature were comprehensive, consisting of an average of 53 indicators per report, and focused on a broad spectrum of HIV care. Peer-reviewed documents retrieved from MEDLINE, EMBASE, and Google Scholar were much more focused on specific aspects of HIV care, with examples including medication adherence, retention in care, and depression among HIV-positive individuals. This focus was reflected in the volume of indicators, which averaged approximately 11 indicators per report.

**Fig 2:**
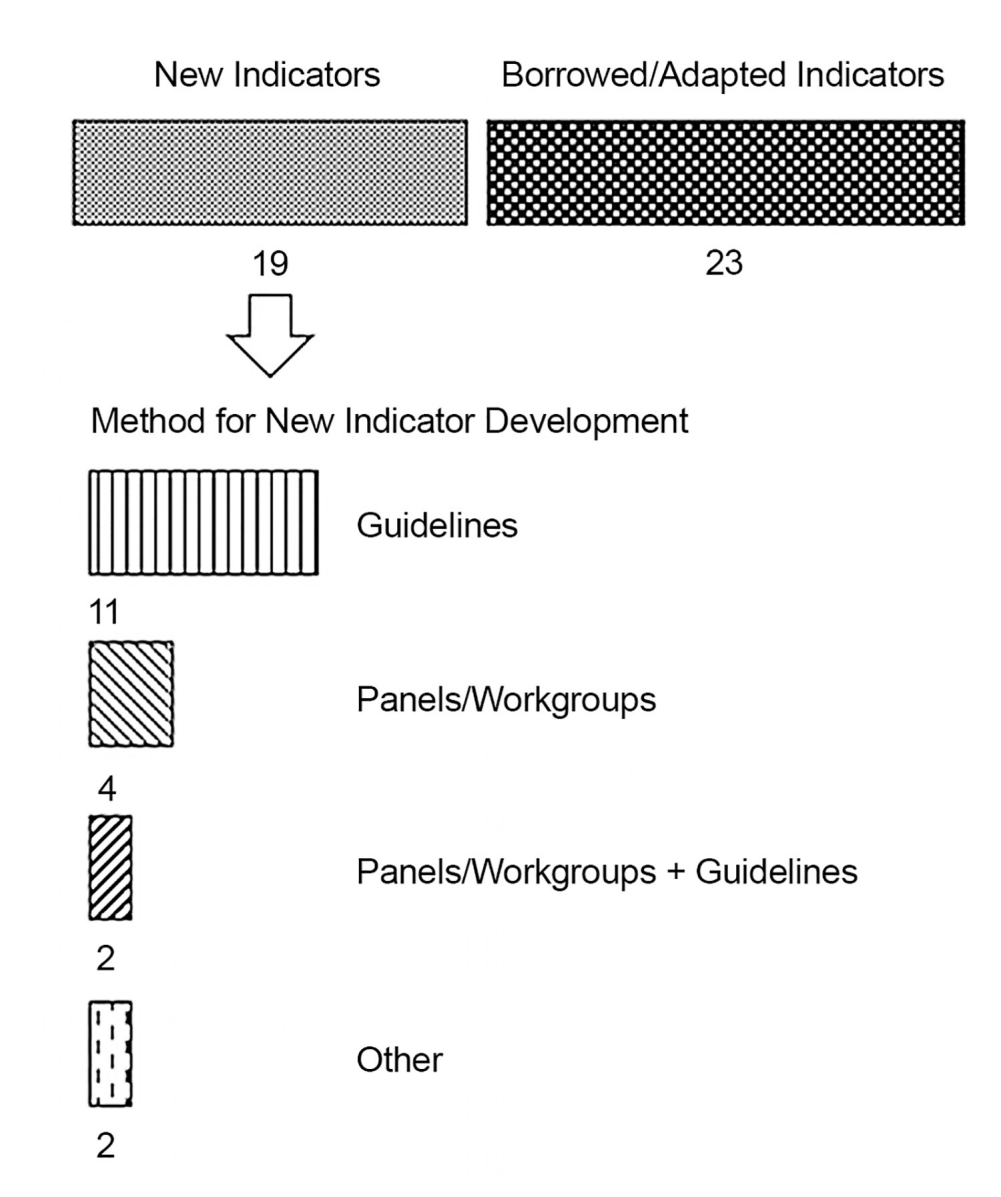
Origins of the HIV performance indicators. The breakdown of how the HIV indicators contained in the publications meeting inclusion criteria of the scoping review were selected in those publications.

The manner in which the indicators were derived for the 47 included documents is outlined in Fig 2. A majority used or adapted existing indicators. The most commonly used source for existing indicators (the source of indicators for 10 of 23 publications) was the United States Department of Health and Human Services (USDHHS) Health Resources and Services Administration (HRSA) HIV/AIDS Bureau’s (HAB) HIV performance measures published in 2012 [13]. The next most popular source for indicators (the New York State Department of Health AIDS Institute, published in 2004 [14]) was used 3 times while a number of other sources were used 1 and 2 times. The publications that derived new indicators used existing practice guidelines, assembled panels or workgroups to guide decisions, or chose a combination of the two strategies (Fig 2).

### Description of existing HIV performance measures

Indicators from the 47 included documents were extracted into a database, resulting in a total of 1184 indicators used since 2000 to evaluate the quality of HIV care (full set available in S4 File). Each of the 1184 indicators was compared against the others to consolidate duplicates and determine the number of unique indicators present in the database. This analysis resulted in 588 distinct indicators for measuring the quality of HIV care. Only 43 of the 588 unique indicators recurred more than 3 times across different sources. The five most recurrent indicators were those attempting to quantify whether an individual was in continuous care (repeated 32 times), whether an individual had been provided PCP prophylaxis (26 times), adequate assessment of CD4 counts (23 times), syphilis testing (18 times), and sufficient number of viral load tests (18 times).

Indicators of patient-reported outcomes were developed in only one of the 19 sources [15] that generated their own new HIV indicators. These patient-reported indicators were adopted by only one other of the 23 documents retrieved in our search that borrowed existing indicators.

### Applicability of HIV Indicators to Primary Care

Each of the 588 distinct extracted indicators was assigned, where applicable, to one of the domains or subdomains of the modified PHC framework. As our focus was on care of people in the primary healthcare setting, we limited our examination of indicators excluding important ones specific to case management, pediatric care, nursing home care, and occupational prophylactic pre-exposure protocols. These exclusions left 505 indicators to assign to the PHC framework. A total of 497 of these 505 unique indicators matched to the modified PHC framework, leaving 8 indicators unmatched. The indicators failing to match the modified PHC framework were indicators quantifying health care system utilization, and indicators relating to hospital policy.

**Fig 3:**
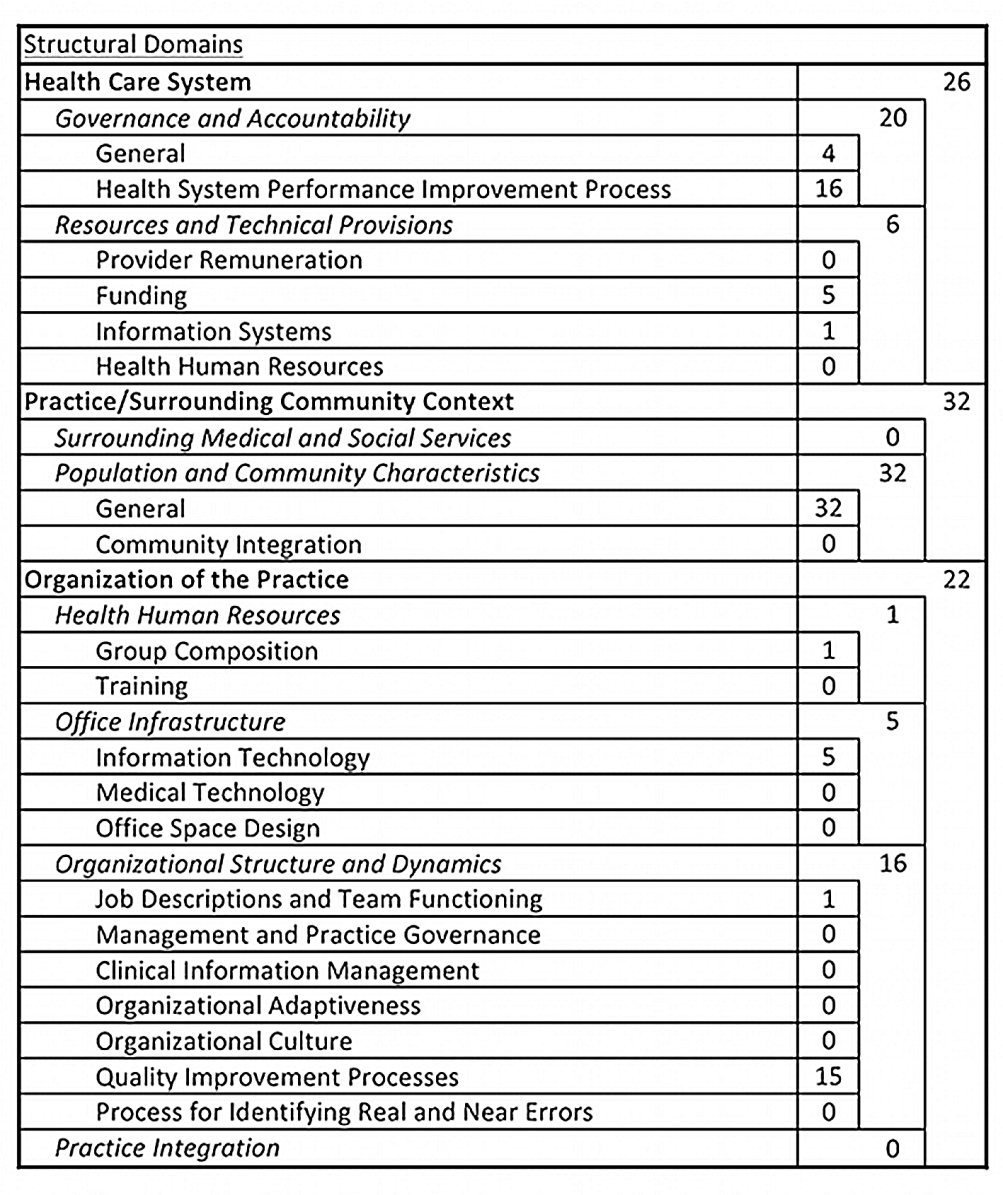
Alignment of HIV performance indicators to the structural aspects of a PHC performance measurement framework.

**Fig 4:**
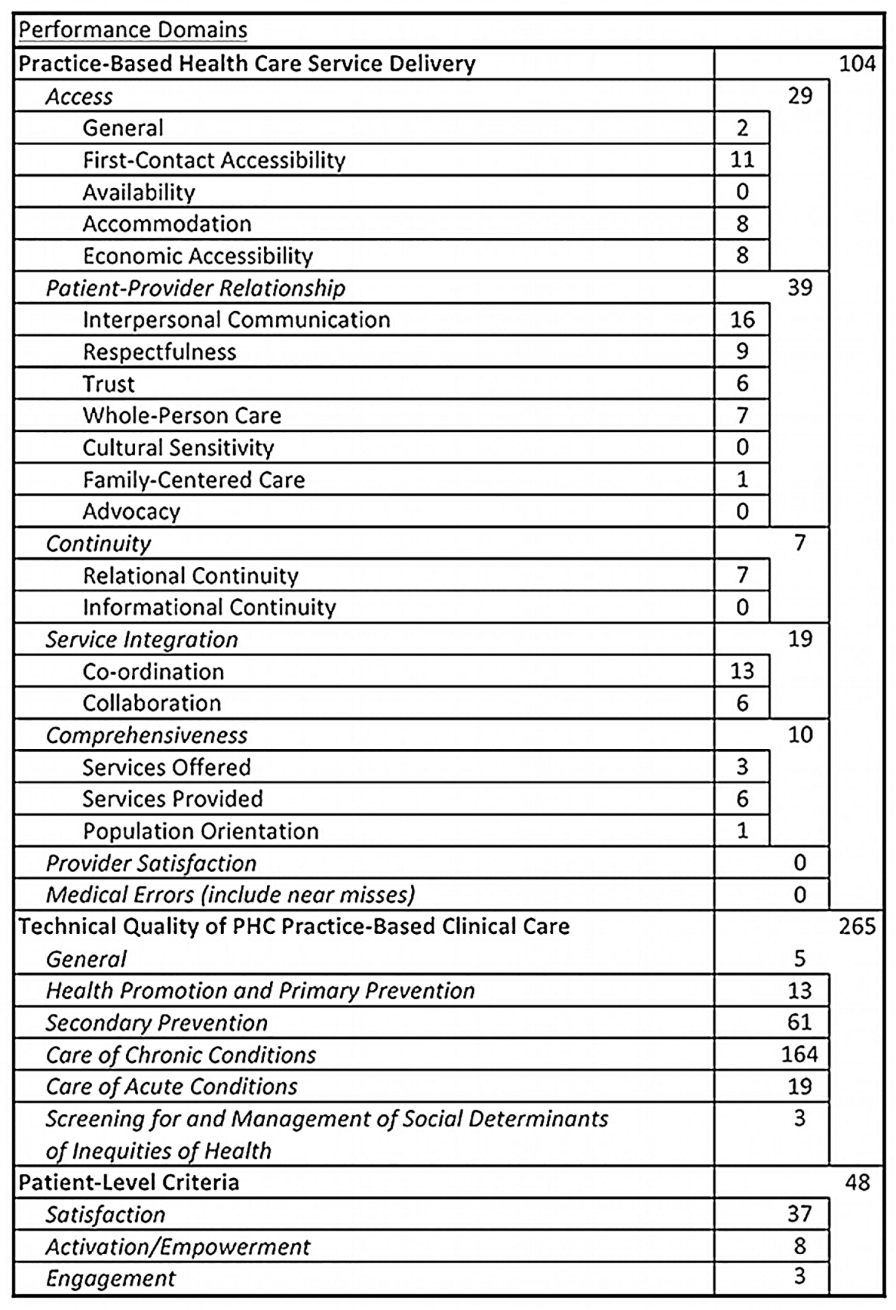
Alignment of HIV performance indicators to the performance aspects of a PHC performance measurement framework.

The modified PHC framework, and how the 497 unique indicators matched to the framework is presented in Figs 3 and 4. The majority of the HIV indicators (417/497 = 84%) focused on the performance elements (Fig 4) as opposed to the structural and organisational elements (Fig 3) of primary care. Within the structural domains, the HIV-specific indicators mostly measured quality/performance improvement processes and population & community characteristics. Fifty-three percent of the unique PHC-relevant indicators were categorized within the technical quality of PHC practice-based clinical care domain. Focusing further within this domain, 61 of the indicators (12% of the total) fell under the ‘secondary prevention’ subdomain and 164 (33% of the total) fell under the ‘care of chronic conditions’ subdomain. Other areas for which the HIV indicators had a significant presence were the two domains (patient-provider relationship and patient satisfaction) that reflect the patient experience of care. These two domains combined accounted for 76 (15% of the total) indicators.

Despite the fact that the PHC-relevant HIV indicators were significantly concentrated in certain aspects of PHC care, we identified at least one indicator applicable to nearly every performance domain of PHC in the framework. Only two domains and four subdomains of care had no HIV indicators match to them. The two domains were medical errors (including near misses) and provider satisfaction. The four subdomains of care without representation; availability, cultural sensitivity, advocacy, and informational continuity, nonetheless fell within domains that were represented in other areas.

## Discussion

The purpose of this work was to identify existing performance indicators to evaluate the quality of care provided to people with HIV in community-based PHC models of care serving this population. Our systematic search identified 47 documents containing performance indicators for evaluating the quality of HIV. Removal of duplicate indicators and matching of indicators to a PHC performance measurement framework resulted in a total of 497 distinct performance indicators highly relevant to PHC. The 497 distinct indicators were heavily focused on the technical aspects of HIV care reflecting the complexity of managing this chronic condition and its initial management within the field of infectious disease. Nonetheless, most reports claiming to provide general HIV care frameworks provided comprehensive sets of indicators highlighting that care for people with HIV is more than optimal disease management. For instance, important elements of high quality PHC over the life span including access, the patient-provider relationship, continuity, integration, and patient satisfaction were represented in the HIV indicators. While our work excluded indicators of care more commonly delivered in settings other than primary healthcare, it was clear from the number of indicators covering aspects of home care, occupational health, and other fields, that the metrics for high quality care of people living with HIV cover the spectrum of care across settings.

Most of the core domains for PHC were populated with indicators from the HIV performance framework indicating the suitability of caring for people with HIV within the PHC system which may be better able to provide the comprehensive and continuous care across the lifespan needed for people with HIV [16]. Certain elements, including provider satisfaction, identifying medical errors, and specific aspects of the patient-provider relationship were not addressed whatsoever in the existing HIV indicators despite evidence that these are important indicators to different stakeholders [17,18]. Importantly, only one source published in 2002 developed indicators which would capture patient-reported outcomes and thus provide information on patients’ experience of care. Advances in chronic disease management models in the last decade, based on the chronic care model [19], recognise the concepts of self-management and patient empowerment which require patient-reported outcomes. Despite the fact that improving the patient experience of care was one of the three priority goals for improving healthcare in the Triple Aim Framework proposed by the Institute for Healthcare Improvement [20], there has been a dearth of ongoing development over the past decade of HIV care performance indicators aimed at capturing the patient’s experience in receiving care. It is concerning that we may not adequately advance our understanding of how the experience of care for people with HIV is impacted by changes in how health services are delivered.

Most of the leading sources of comprehensive performance indicators for HIV care used a process involving a review of the scientific evidence for the standard of care and expert review. For performance indicators to contribute or support ongoing health system transformation for better care for people with HIV, the indicators need to be relevant to the many stakeholders contributing to change. The strong match of HIV indicators to a PHC performance framework suggests there is high relevance. However ongoing development of performance indicators, recognising the growing role PHC may play in the care of people with HIV, should include PHC providers and patients cared for in the PHC system. Further, performance indicators may measure and report on provider, practice, or population level performance with different target audiences for such information. The spread of indicators across the PHC performance framework showed that population level measures such as population and community characteristics, as well as practice and provider specific-measures are all important to high functioning primary healthcare for patients with complex chronic conditions like HIV. While many of the indicators would be relevant across all three levels of measurement, it would be important in measurement initiatives to ensure that the stakeholders necessary to improve primary healthcare for people living with HIV had the information most relevant to them.

With comprehensive care as a goal for people living with a complex chronic condition like HIV, updating of performance indicators should not just focus on emerging evidence on the technical quality of care such as medication management or optimal secondary prevention practices. While these are particularly important elements of care for complex conditions and need to be aligned with constantly evolving evidence and practice guidelines, ensuring that best practices in other domains such as the organisation of care, patient-provider relationship, patient safety and medical errors, continues to be integrated into the performance goals for people with HIV must be a goal of comprehensive performance frameworks seeking to guide health system improvement.

## Conclusion

Existing performance frameworks for the care of people with HIV provide a comprehensive set of indicators that align well with a primary healthcare performance framework despite some important elements of care not well covered by existing indicators. As more people with HIV are cared for in the PHC system or shared-care models involving specialty care and PHC, the framework created by this study organising performance indicators by PHC performance domain can serve as a base to select indicators to evaluate the care for people with HIV.

## Supporting Information

S1 File PRISMA Checklist. A list of the components of a systematic review, where in the manuscript each item is located, and, where applicable, how the items were adapted for the scoping review.
(DOCX)

S2 File Search strategy. A description of the strategy used to search the Medline and Embase databases as well as the Google and Google Scholar search engines.
(DOCX)

S3 File HIV performance measurement documents. A list of the articles and reports included in this study from which HIV performance indicators were drawn and analyzed.
(DOCX)

S4 File HIV performance indicators. A database file complete with each of the 1184 indicators extracted, the primary care performance domain it was matched to, its source, where the indicator was derived from (if different from the source), whether the indicator is unique or duplicated, and if duplicated, which indicator it was matched to.
(XLSX)
