# Supplementary material for: Measures of Quality of Care for People with HIV: A Scoping Review of Performance Indicators for Primary Care
Source: PLoS One. 2015 Sep 28;10(9):e0136757. doi: 10.1371/journal.pone.0136757 (PMC4586139; doi:10.1371/journal.pone.0136757)
Supplement: S2 File — A description of the strategy used to search the Medline and Embase databases as well as the Google and Google Scholar search engines. (DOCX) [file pone.0136757.s002.docx]

Medline Search Strategy

1. *HIV/ or *Acquired Immunodeficiency Syndrome/ or *HIV Infections/

2. quality indicator*.mp.

3. quality measure*.mp.

4. performance indicator*.mp.

5. performance measure*.mp.

6. benchmark*.mp.

7. 2 or 3 or 4 or 5 or 6

8. 1 and 7

9. limit 8 to (english language and yr="2000 -Current")

Embase Search Strategy

1. quality indicator*.mp.

2. quality measure*.mp.

3. performance indicator*.mp.

4. performance measure*.mp.

5. benchmark*.mp.

6. 1 or 2 or 3 or 4 or 5

7. *Human immunodeficiency virus infection/ or *Human immunodeficiency virus infected patient/ or *Human immunodeficiency virus/ or *acquired immune deficiency syndrome/

8. 6 and 7

9. limit 8 to (english language and yr="2000 -Current")

Google/Google Scholar Search Strategy

(("quality indicator" OR "quality measure" OR "performance indicator" OR "performance measure"))((AIDS OR "acquired immunodeficiency syndrome" OR HIV OR "human immunodeficiency virus")
