# Supplementary material for: Measures of Quality of Care for People with HIV: A Scoping Review of Performance Indicators for Primary Care
Source: PLoS One. 2015 Sep 28;10(9):e0136757. doi: 10.1371/journal.pone.0136757 (PMC4586139; doi:10.1371/journal.pone.0136757)
Supplement: S3 File — A list of the articles and reports included in this study from which HIV performance indicators were drawn and analyzed. (DOCX) [file pone.0136757.s003.docx]

| **First Author/Publisher** | **Year** | **Title** | **Ref** |
| --- | --- | --- | --- |
| Agency for Healthcare Research and Quality | 2013 | National Quality Measures Clearinghouse | [1] |
| American Medical Association | 2010 | HIV Quality Measures: National Development And in Kaiser Permanente | [2] |
| Asch, S.M. | 2004 | Symptom-based framework for assessing quality of HIV care | [3] |
| Backus, L.I. | 2010 | National quality forum performance measures for HIV/AIDS care: the Department of Veterans Affairs' experience | [4] |
| Chow, W. | 2012 | Improved quality of HIV care over time among participants in a national quality improvement initiative | [5] |
| Connecticut Department of Public Health | 2011 | Health Care and Support Services Quality Management Program | [6] |
| Dang, B.N. | 2013 | Examining the Link between Patient Satisfaction and Adherence to HIV Care: A Structural Equation Model | [7] |
| Ding, L. | 2008 | The quality of care received by HIV patients without a primary provider | [8] |
| Gross, P.A. | 2000 | Performance measures for guidelines on preventing opportunistic infections in patients infected with human immunodeficiency virus | [9] |
| Hall, H.I. | 2012 | Retention in care of adults and adolescents living with HIV in 13 U.S. areas | [10] |
| Hirschorn, L.R. | 2009 | Reported care quality in federal Ryan White HIV/AIDS Program supported networks of HIV/AIDS care | [11] |
| HIV Guidelines.org | 2013 | NY State Department of Health AIDS Institute HIV Quality of Care Program - Adult Indicators | [12] |
| HIVQUAL-US | 2011 | HIVQUAL-US Annual Data Report | [13] |
| Horberg, M. | 2011 | HIV quality performance measures in a large integrated health care system | [14] |
| Horberg, M.A. | 2010 | Development of national and multiagency HIV care quality measures | [15] |
| HRSA HIV/AIDS Bureau | 2012 | Health Resources and Services Administration HIV/AIDS Bureau Performance Measures | [16] |
| Institute for Healthcare Improvement | 2012 | Institute for Healthcare Improvement Measures | [17] |
| Institute of Medicine | 2012 | Monitoring HIV Care in the United States: Indicators and Data Systems | [18] |
| Kalichman, S.C. | 2009 | A simple single-item rating scale to measure medication adherence: further evidence for convergent validity | [19] |
| Kerr, C.A. | 2012 | HIV quality of care assessment at an academic hospital: outcomes and lessons learned | [20] |
| Kitahata, M.M. | 2003 | Electronic human immunodeficiency virus (HIV) clinical reminder system improves adherence to practice guidelines among the University of Washington HIV Study Cohort | [21] |
| Koethe, J.R. | 2008 | Physician specialization and women's primary care services in an urban HIV clinic | [22] |
| Korthuis, P.T. | 2011 | Improving adherence to HIV quality of care indicators in persons with opioid dependence: the role of buprenorphine | [23] |
| Korthuis, P.T. | 2004 | Quality of HIV Care within the Veterans Affairs Health System: A Comparison Using Outcomes from the HIV Cost and Services Utilization Study | [24] |
| Korthuis, P.T. | 2012 | Unhealthy Alcohol and Illicit Drug Use Are Associated With Decreased Quality of HIV Care | [25] |
| Landon, B.E. | 2004 | Effects of a quality improvement collaborative on the outcome of care of patients with HIV infection: the EQHIV study | [26] |
| Lima, V.D. | 2012 | Development and validation of a composite programmatic assessment tool for HIV therapy | [27] |
| Mugavero, M.J. | 2010 | From access to engagement: measuring retention in outpatient HIV clinical care | [28] |
| National Quality Forum | 2013 | NQF Quality Positioning System | [29] |
| New York Dep. Of Health AIDS Institute | 2008 | Guidline-based Quality Indicators for HIV Care | [30] |
| New York Department of Health AIDS Institute | 2012 | HIVQUAL-US: ADULT HIV AMBULATORY CARE QUALITY OF CARE INDICATOR DEFINITIONS | [31] |
| New York Department of Health AIDS Institute | 2002 | Patient Satisfaction Survey for HIV Ambulatory Care | [32] |
| Pyne, J.M. | 2008 | Quality indicators for depression care in HIV patients | [33] |
| Rebeiro, P. | 2013 | Retention among North American HIV-infected persons in clinical care, 2000-2008 | [34] |
| Rothman, J. | 2007 | Co-located substance use treatment and HIV prevention and primary care services, New York State, 1990-2002: a model for effective service delivery to a high-risk population | [35] |
| Ryan White Hartford organization | 2013 | Hartford Outpatient Ambulatory Care Services Performance Measures | [36] |
| Santa Clara County Public Health | 2012 | HIV Services Quality Management Plan | [37] |
| Solomon, L. | 2005 | Managed care for AIDS patients: is bigger better? | [38] |
| Steinbock, C. | 2011 | Performance Measurement in HIV Care | [39] |
| Strike, C. | 2011 | Guidelines for better harm reduction: evaluating implementation of best practice recommendations for needle and syringe programs (NSPs) | [40] |
| Torres, G.W. | 2009 | HIV testing and referral to care in U.S. hospitals prior to 2006: results from a national survey | [41] |
| US Dept. of Health and Human Services -  Health Resources and Services Administration | 2008 | HAB HIV Core Clinical Performance Measures for Adult/Adolescent Clients | [42] |
| Valenti, W.M. | 2000 | HIV, managed care, and outcomes | [43] |
| Virga, P.H. | 2012 | Electronic health information technology as a tool for improving quality of care and health outcomes for HIV/AIDS patients | [44] |
| Wilson, I.B. | 2007 | Correlations among measures of quality in HIV care in the United States: cross sectional study | [45] |
| Wilson, I.B. | 2005 | Quality of HIV care provided by nurse practitioners, physician assistants, and physicians | [46] |
| Yehia, B.R. | 2012 | Comparing different measures of retention in outpatient HIV care | [47] |

Reference List

1. Agency for Healthcare Research and Quality. **National Quality Measures Clearinghouse.** 2013. Agency for Healthcare Research and Quality. 24-9-2013.

2. Horberg M: *HIV Quality Measures: National Development And in Kaiser Permanente.* 2010.

3. Asch SM, Fremont AM, Turner BJ, Gifford A, McCutchan JA, Mathews WM, Bozzette SA, Shapiro MF: **Symptom-based framework for assessing quality of HIV care.** *Int J Qual Health Care* 2004, **16:**41-50.

4. Backus LI, Boothroyd DB, Phillips BR, Belperio PS, Halloran JP, Valdiserri RO, Mole LA: **National quality forum performance measures for HIV/AIDS care: the Department of Veterans Affairs' experience.** *Arch Intern Med* 2010, **170:**1239-1246.

5. Chow W, Hirschhorn LR, Ng DW, Wells CG, Schneider KL, Agins BD: **Improved quality of HIV care over time among participants in a national quality improvement initiative.** *J Health Care Poor Underserved* 2012, **23:**67-80.

6. Connecticut Department of Public Health: *Health Care and Support Services Quality Management Program.* 2011.

7. Dang BN, Westbrook RA, Black WC, Rodriguez-Barradas MC, Giordano TP: **Examining the link between patient satisfaction and adherence to HIV care: a structural equation model.** *PLoS One* 2013, **8:**e54729.

8. Ding L, Landon BE, Wilson IB, Hirschhorn LR, Marsden PV, Cleary PD: **The quality of care received by HIV patients without a primary provider.** *AIDS Care* 2008, **20:**35-42.

9. Gross PA, Asch S, Kitahata MM, Freedberg KA, Barr D, Melnick DA, Bozzette SA: **Performance measures for guidelines on preventing opportunistic infections in patients infected with human immunodeficiency virus.** *Clin Infect Dis* 2000, **30 Suppl 1:**S85-S93.

10. Hall HI, Gray KM, Tang T, Li J, Shouse L, Mermin J: **Retention in care of adults and adolescents living with HIV in 13 U.S. areas.** *J Acquir Immune Defic Syndr* 2012, **60:**77-82.

11. Hirschhorn LR, Landers S, McInnes DK, Malitz F, Ding L, Joyce R, Cleary PD: **Reported care quality in federal Ryan White HIV/AIDS Program supported networks of HIV/AIDS care.** *AIDS Care* 2009, **21:**799-807.

12. New York Department of Health AIDS Institute, Johns Hopkins University Division of Infectious Diseases. **HIV Clinical Resource.** 2004. New York Department of Health AIDS Institute. 24-9-2013.

13. HIVQUAL-US: *HIVQUAL-US Annual Data Report.* 2011.

14. Horberg M, Hurley L, Towner W, Gambatese R, Klein D, Antoniskis D, Weinberg W, Kadlecik P, Remmers C, Dobrinich R et al.: **HIV quality performance measures in a large integrated health care system.** *AIDS Patient Care STDS* 2011, **25:**21-28.

15. Horberg MA, Aberg JA, Cheever LW, Renner P, O'Brien KE, Asch SM: **Development of national and multiagency HIV care quality measures.** *Clin Infect Dis* 2010, **51:**732-738.

16. HRSA HIV/AIDS Bureau. **Health Resources and Services Administration HIV/AIDS Bureau Performance Measures.** 1-6-2012. AIDS Education and Training Center National Resource Center.

17. Institute for Healthcare Improvement. **Institute for Healthcare Improvement Measures.** 2012. Institute for Healthcare Improvement. 24-9-2013.

18. *Monitoring HIV Care in the United States: Indicators and Data Systems.* The National Academies Press; 2012.

19. Kalichman SC, Amaral CM, Swetzes C, Jones M, Macy R, Kalichman MO, Cherry C: **A simple single-item rating scale to measure medication adherence: further evidence for convergent validity.** *J Int Assoc Physicians AIDS Care (Chic )* 2009, **8:**367-374.

20. Kerr CA, Neeman N, Davis RB, Schulze J, Libman H, Markson L, Aronson M, Bell SK: **HIV quality of care assessment at an academic hospital: outcomes and lessons learned.** *Am J Med Qual* 2012, **27:**321-328.

21. Kitahata MM, Dillingham PW, Chaiyakunapruk N, Buskin SE, Jones JL, Harrington RD, Hooton TM, Holmes KK: **Electronic human immunodeficiency virus (HIV) clinical reminder system improves adherence to practice guidelines among the University of Washington HIV Study Cohort.** *Clin Infect Dis* 2003, **36:**803-811.

22. Koethe JR, Moore RD, Wagner KR: **Physician specialization and women's primary care services in an urban HIV clinic.** *AIDS Patient Care STDS* 2008, **22:**373-380.

23. Korthuis PT, Fiellin DA, Fu R, Lum PJ, Altice FL, Sohler N, Tozzi MJ, Asch SM, Botsko M, Fishl M et al.: **Improving adherence to HIV quality of care indicators in persons with opioid dependence: the role of buprenorphine.** *J Acquir Immune Defic Syndr* 2011, **56 Suppl 1:**S83-S90.

24. Korthuis PT, Anaya HD, Bozzette SA, Brinkerhoff C, Mancewicz M, Wang M, Asch S: **Quality of HIV Care within the Veterans Affairs Health System: A Comparison Using Outcomes from the HIV Cost and Services Utilization Study.** *Journal of Clinical Outcomes* 2004, **11:**765-774.

25. Korthuis PT, Fiellin DA, McGinnis KA, Skanderson M, Justice AC, Gordon AJ, Doebler DA, Asch SM, Fiellin LE, Bryant K et al.: **Unhealthy alcohol and illicit drug use are associated with decreased quality of HIV care.** *J Acquir Immune Defic Syndr* 2012, **61:**171-178.

26. Landon BE, Wilson IB, McInnes K, Landrum MB, Hirschhorn L, Marsden PV, Gustafson D, Cleary PD: **Effects of a quality improvement collaborative on the outcome of care of patients with HIV infection: the EQHIV study.** *Ann Intern Med* 2004, **140:**887-896.

27. Lima VD, Le A, Nosyk B, Barrios R, Yip B, Hogg RS, Harrigan PR, Montaner JS: **Development and validation of a composite programmatic assessment tool for HIV therapy.** *PLoS One* 2012, **7:**e47859.

28. Mugavero MJ, Davila JA, Nevin CR, Giordano TP: **From access to engagement: measuring retention in outpatient HIV clinical care.** *AIDS Patient Care STDS* 2010, **24:**607-613.

29. National Quality Forum. **NQF Quality Positioning System.** 2013. National Quality Forum. 24-9-2013.

30. New York Department of Health AIDS Institute, Health Resources and Services Administration HIV/AIDS Bureau: *Guideline-based Quality Indicators for HIV Care.* New York, NY; 2008.

31. New York Department of Health AIDS Institute, US Department of Health and Human Services, HRSA/HAB/Division of Community Based Programs: *HIVQUAL-US: ADULT HIV AMBULATORY CARE QUALITY OF CARE INDICATOR DEFINITIONS.* 2012.

32. New York Department of Health AIDS Institute: *Patient Satisfaction Survey for HIV Ambulatory Care.* 2002.

33. Pyne JM, Asch SM, Lincourt K, Kilbourne AM, Bowman C, Atkinson H, Gifford A: **Quality indicators for depression care in HIV patients.** *AIDS Care* 2008, **20:**1075-1083.

34. Rebeiro P, Althoff KN, Buchacz K, Gill J, Horberg M, Krentz H, Moore R, Sterling TR, Brooks JT, Gebo KA et al.: **Retention among North American HIV-infected persons in clinical care, 2000-2008.** *J Acquir Immune Defic Syndr* 2013, **62:**356-362.

35. Rothman J, Rudnick D, Slifer M, Agins B, Heiner K, Birkhead G: **Co-located substance use treatment and HIV prevention and primary care services, New York State, 1990-2002: a model for effective service delivery to a high-risk population.** *J Urban Health* 2007, **84:**226-242.

36. City of Hartford: *Outpatient Ambulatory Care Service Performance Measures for 2012-2013.* 2013.

37. Santa Clara County Public Health, John Snow I: *HIV Services Quality Management Plan; San Jose, CA (Santa Clara County) Transitional Grant Area.* 2013.

38. Solomon L, Flynn C, Lavetsky G: **Managed care for AIDS patients: is bigger better?** *J Acquir Immune Defic Syndr* 2005, **38:**342-347.

39. Steinbock C: *Performance Measurement in HIV Care.* 2011.

40. Strike C, Watson TM, Lavigne P, Hopkins S, Shore R, Young D, Leonard L, Millson P: **Guidelines for better harm reduction: evaluating implementation of best practice recommendations for needle and syringe programs (NSPs).** *Int J Drug Policy* 2011, **22:**34-40.

41. Torres GW, Yonek J, Pickreign J, Whitmore H, Hasnain-Wynia R: **HIV testing and referral to care in U.S. hospitals prior to 2006: results from a national survey.** *Public Health Rep* 2009, **124:**400-408.

42. Health Resources and Services Administration. **HAB HIV Performance Measures: Update on the HIV/AIDS Bureau's Performance Measure Portfolio.** 2013. U.S. Department of Health and Human Services. 24-9-2013.

43. Valenti WM: **HIV, managed care, and outcomes.** *AIDS Read* 2000, **10:**274-278.

44. Virga PH, Jin B, Thomas J, Virodov S: **Electronic health information technology as a tool for improving quality of care and health outcomes for HIV/AIDS patients.** *Int J Med Inform* 2012, **81:**e39-e45.

45. Wilson IB, Landon BE, Marsden PV, Hirschhorn LR, McInnes K, Ding L, Cleary PD: **Correlations among measures of quality in HIV care in the United States: cross sectional study.** *BMJ* 2007, **335:**1085.

46. Wilson IB, Landon BE, Hirschhorn LR, McInnes K, Ding L, Marsden PV, Cleary PD: **Quality of HIV care provided by nurse practitioners, physician assistants, and physicians.** *Ann Intern Med* 2005, **143:**729-736.

47. Yehia BR, Fleishman JA, Metlay JP, Korthuis PT, Agwu AL, Berry SA, Moore RD, Gebo KA: **Comparing different measures of retention in outpatient HIV care.** *AIDS* 2012, **26:**1131-1139.
